# A Mobile App (BEDSide Mobility) to Support Nurses’ Tasks at the Patient's Bedside: Usability Study

**DOI:** 10.2196/mhealth.9079

**Published:** 2018-03-21

**Authors:** Frederic Ehrler, Thomas Weinhold, Jonathan Joe, Christian Lovis, Katherine Blondon

**Affiliations:** ^1^ Division of Medical Information Sciences University Hospitals of Geneva Geneva Switzerland; ^2^ Biomedical & Health Informatics University of Washington Seattle, WA United States; ^3^ Faculty of Medicine University Of Geneva Geneva Switzerland; ^4^ Department of General Internal Medicine University Hospitals of Geneva Geneva Switzerland

**Keywords:** clinical information system, mobile health, usability testing

## Abstract

**Background:**

The introduction of clinical information systems has increased the amount of clinical documentation. Although this documentation generally improves patient safety, it has become a time-consuming task for nurses, which limits their time with the patient. On the basis of a user-centered methodology, we have developed a mobile app named BEDSide Mobility to support nurses in their daily workflow and to facilitate documentation at the bedside.

**Objective:**

The aim of the study was to assess the usability of the BEDSide Mobility app in terms of the navigation and interaction design through usability testing.

**Methods:**

Nurses were asked to complete a scenario reflecting their daily work with patients. Their interactions with the app were captured with eye-tracking glasses and by using the think aloud protocol. After completing the tasks, participants filled out the system usability scale questionnaire. Descriptive statistics were used to summarize task completion rates and the users’ performance.

**Results:**

A total of 10 nurses (aged 21-50) participated in the study. Overall, they were satisfied with the navigation, layout, and interaction design of the app, with the exception of one user who was unfamiliar with smartphones. The problems identified were related to the ambiguity of some icons, the navigation logic, and design inconsistency.

**Conclusions:**

Besides the usability issues identified in the app, the participants’ results do indicate good usability, high acceptance, and high satisfaction with the developed app. However, the results must be taken with caution because of the poor ecological validity of the experimental setting.

## Introduction

### Background

The introduction of clinical information system in hospitals has impacted the workflow of nurses in several ways. Despite positive consequences in terms of patient safety and quality of care [1-3], the use of such systems has also resulted in an increase in the documentation workload with a subsequent shift of nursing activity from the patient bedside to the computer [3-5]. Studies have reported that up to 30% of daily workload was spent on documentation [6]. Until recently, all this clinical documentation was performed on desktop computers, which keeps nurses away from the patient bedside [7], induces transcription errors [8], and creates a delay in the availability of collected data within the electronic health record (EHR). To some extent, this problem has been addressed with the use of wireless networks and computers on wheels (COWs) [9], but mobility can be further increased by the use of smartphones and mobile apps [10,11].

The transition from a system designed for desktop computer to a small smartphone screen is a complex task. Careful attention must be given to choosing the most useful functionalities of such systems and for the design of the user interface [12]. Otherwise, this transition can easily lead to unexpected failures such as an increased number of input errors [13], loss of data, or decreased efficiency, as well as user frustration, and discontent [14-16].

In this paper, we have presented the usability testing of a mobile app named BEDSide Mobility, which was developed to support nursing workflow at the patient bedside.

### The Current Intervention Process

The University Hospitals of Geneva is a consortium of public hospitals in Geneva, Switzerland. It provides primary, secondary, tertiary, and outpatient care for the whole region with 50,000 inpatients and 950,000 outpatient visits a year.

Patient data are managed by a clinical information system (CIS) that possesses most features of modern CIS such as computerized physician order entry, clinical pathways, care management, laboratory, imaging, etc. One of its modules supports the work of nurses by providing a list of their daily tasks. These tasks cover a large range of interventions such as assistance for bathing, drug administration, or wound care. Interventions are planned by nurses, either as a nursing-based task or in response to a physician’s prescription. They are defined by several parameters such as their type, date and time of planning, and the start and end dates. The task list module of the CIS allows visualization of a patient’s intervention list in several ways such as by shift, by type of task, by room, and by nurse, etc.

There is no clear guidance regarding the way nurses have to manage their list of interventions. However, most of the time, they take a printout of the list of interventions they have to perform during the day at the beginning of their shift. Then, they perform their care following the instructions that are given on the list. Every time nurses perform one of these interventions, they tick the intervention on their printouts, indicating that the task has been completed. At a later time of day, they enter these validations and other gathered information into the CIS. This process has only partly changed even with the implementation of COWs in the wards. Indeed, the COW trolley is not always adapted to the room setting, preventing access to the CIS at the bedside.

### The BEDSide Mobility App

The introduction of the BEDSide Mobility app aims at suppressing media disruptions and at enabling the management and documentation of interventions at the patients' bedside [17]. The app was developed based on a user-centered design approach [18]. The iterative development included focus group sessions and informal usability evaluations at different times in the agile development cycle as well as a heuristic evaluation of an advanced version of the prototype. This allowed a continuous improvement of the app.

**Figure 1:**
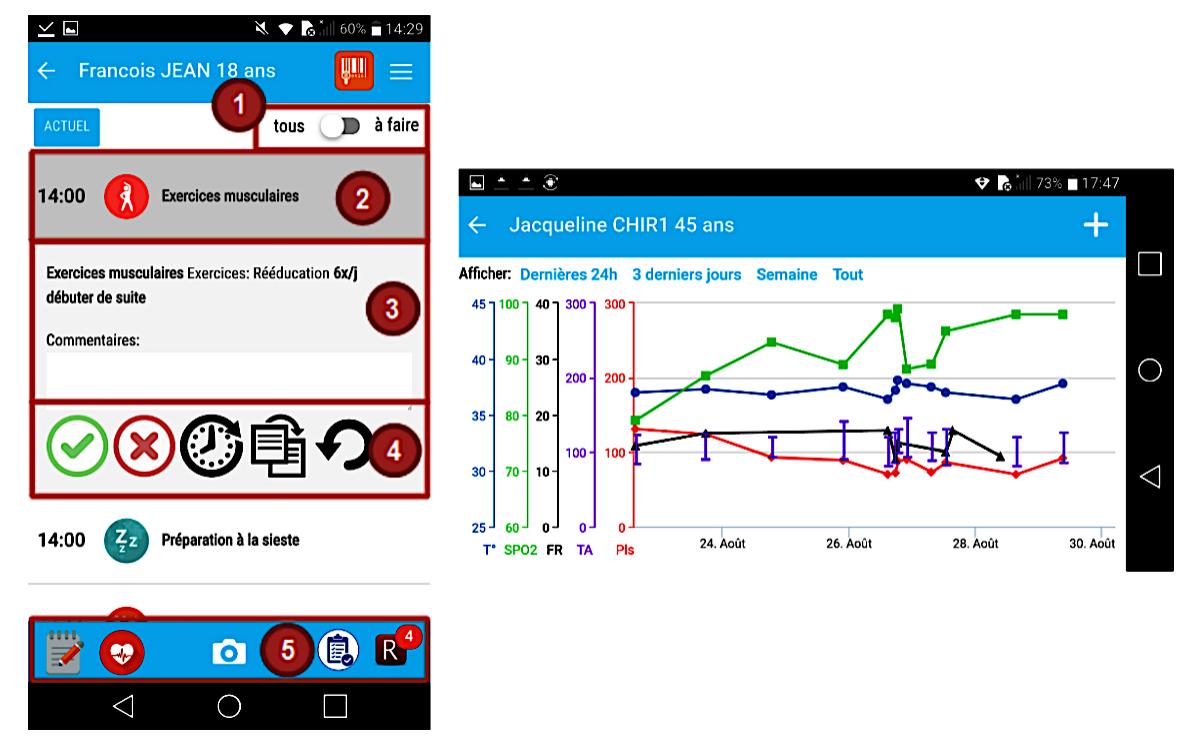
Main screen and vital sign screen of the BEDSide Mobility app.

The tool supports the entire nurse workflow [19,20]. Nurses start by selecting the rooms and patients under their responsibility during their shift and access their patient’s charts either by selecting a ward with a patient list or by scanning a quick response (QR) code on the patient’s hospital bracelets with the smartphone camera [21]. In the patient charts, the nurses have access to all the planned interventions (Figure 1). These interventions are presented in chronological order, starting at the current time of app use (Figure 1, point 2). Interventions of similar nature (different types of medication, for example) are grouped together for easier readability. Interventions can be validated with rapid swipe motions but can also be modified, delayed, or repeated (Figure 1, point 4). These functions are often used when documenting in the EHR. Each patient chart also includes administrative patient information (identity, age, length of hospital stay) and clinical data on the current hospitalization such as comorbidities and daily nursing objectives. These components were included to provide support for the handoff process [22-24]. Vital signs and clinical scores can be entered and visualized easily in the app as data entry is directly available from the task list to facilitate usability. Users can also add vital signs and clinical score results through the data visualization screen, even when they are not planned tasks (Figure 1, point 5). The vital signs graph is similar to the EHR graphs for familiarity and easier readability [25,26]. The “pro re nata” (PRN) use medication (or medication “as needed”) is available in another part of the app, with indications of prescribed doses and frequencies [27]. It also records when these PRN medications have been administered during the past 24 hours.

Before the usability study, a heuristic evaluation was performed on the app using Neilsen's usability heuristics [28]. The heuristic review identified usability issues such as problems with confusing and unclear labels as well as icons, unexpected behavior, confusing navigation, and consistency issues. Users were not sure where an icon or label would take them and were frustrated by iconography or patterns that did not generate the same behavior in the app. Alternatively, similar tasks required the user to have different input or actions to accomplish them. Remedies addressing these identified usability issues were implemented before the lab tests reported in this paper.

## Methods

### Study Design

The usability test consisted of a human-computer interaction evaluation, which focused on an outcome quality, user perception, and user performance in the laboratory setting. It consisted of the completion of 10 goal-oriented tasks by targeted end users [29].

The tests were carried out between August 8, 2016 and August 12, 2016 in the Evalab, a medical informatics lab room, which was arranged to simulate a hospital room. It contained a bed with a mannequin that had a patient identification bracelet with a QR code. The tests were run on a Samsung Galaxy Xcover 3 with a 4.5-inch screen size and a resolution of 480 x 800 pixels and an Android OS V4.4.4 (KitKat).

To record the participants’ interactions, participants wore eye-tracking glasses (ETG by SMI, sampling rate 30 Hz). Although the glasses are less discreet than peripheral cameras, they have the advantage of allowing us to also see the smartphone screens clearly, rather than just the users’ actions. Participants were asked to perform the scenario and tasks, which are described below. To gain a deeper insight into the behavior and the considerations of the users, participants were asked to describe their actions by using the “think aloud” protocol [30].

### Scenarios

Two scenarios, a surgical and a medical one, were created and validated by a surgical head nurse, a medical head nurse, and a physician. Both scenarios intended to recreate a realistic situation where nurses would have to use the app to interact with the clinical information system. During these scenarios, the participants had to interact with the app to complete 10 typical tasks reflecting the most frequent actions in the nurses' daily work. This allowed us to validate most of the functionalities of the tool. It is important to highlight that the actions requested by the nurses were strictly limited to the interaction with the app and therefore their completion of a task only required the app. To have a high level of realism and to reflect the actual workflow in the different wards, drug names, dosages, etc, were adapted for surgical and medical unit settings. Although the details of the tasks differed between the scenarios, performing these tasks required the use of the same app functionalities, enabling us to combine the results of the scenarios.

The following list provides an overview of the requested interactions according to the tasks of the surgical and medical scenario:

1. Identification of the patient by scanning the QR code on his bracelet
2. Review of the interventions performed during the night
3. Stating the necessary interventions for the medication rounds (3a); validating the administration of drugs (3b); removing the validation for breakfast (3c)
4. Postponing (4a) and duplication (4b) of an intervention
5. Validating the start of an intravenous (IV) drug (5a), checking the PRN painkillers, administration of a dose of painkiller, and validation of this action in the app (5b)
6. Indication of the elapsed volume for the intravenous drug (6a), documentation of the patient's pain level (6b)
7. Documentation that the patient refused to eat his dinner
8. Completing a Braden scale (8a) and taking a photo (8b)
9. Listing of remaining interventions to be completed before the end of the working shift
10. Validation of all open interventions and log out

### Participants

The study participants were recruited in several medical and surgical wards of our hospital. The only inclusion criterion was to have more than 6 months experience (clinical experience and experience with our EHR).

### Procedure

The participants were invited to an individual session. After signing the consent form, the participants filled out a short questionnaire about demographics, satisfaction with the CIS, and their familiarity with smartphones and mobile apps. Subsequently, the test manager presented the main functionalities of the tool to the participants in 5 to 10 min. After setting up and calibrating the eye-tracking system, participants began the evaluation. The test manager gave the participants a printout of the scenario with the list of 10 goal-oriented corresponding tasks. The participants were asked to complete the tasks by themselves and to think aloud if possible. The test manager did not offer any help during the task execution. This aimed at minimizing any disruptions of the spontaneous thoughts of the participants as well as to avoid bias on the results.

After completing the goal-oriented tasks, the participants filled out a paper version of the system usability scale questionnaire (SUS). SUS is a standardized and simple tool to get a global view of the participants’ subjective assessment of usability based on 10 questions [31]. For this study, a French translation of the original items was used.

### Data Analysis

The videos from the test sessions that were created with the ETG were imported into Techsmith Morae. We used mixed methods for the analysis with quantitative analyses of the success rates and task duration and a qualitative analysis of the problems the nurses encountered.

Two independent evaluators analyzed the video recordings for the duration of the tasks. The timer began after the participant read the task instructions and ended when the participant performed the correct action or gave the proper answer to the final task. The reported time was computed as the mean between the two calculations. In case of a disagreement larger than 10%, a third evaluator helped to reach a consensus.

The analysis of the SUS score was conducted according to the scoring strategy of Brooke [31]. The score for each item ranges from 0 to 4. With regard to the positively worded items (1, 3, 5, 7, 9) the score contribution is computed as the scale position minus 1. For the negatively worded items (2, 4, 6, 8, 10), the contribution is computed as 5 minus the scale position. Afterwards, the sum of the scores is multiplied by 2.5 to get the overall value of SUS ranging from 0 to 100 [6].

## Results

### Participants

In total, 10 nurses participated in the study. Sixty percent (6/10) of the nurses had more than 5 years of professional experience, with the rest (4/10) having between 1 to 5 years of experience. This duration corresponded with their experience with the use of the institutional CIS since it has existed for more than 10 years. Overall, the nurses were satisfied with the CIS. Sixty percent (6/10) indicated that they were very satisfied (20%) or satisfied with the CIS (40%). The remaining 40% (4/10) were rather satisfied with the system. Table 1 provides more detailed descriptions of the participants.

### Visual and Interaction Design

**Figure 2:**
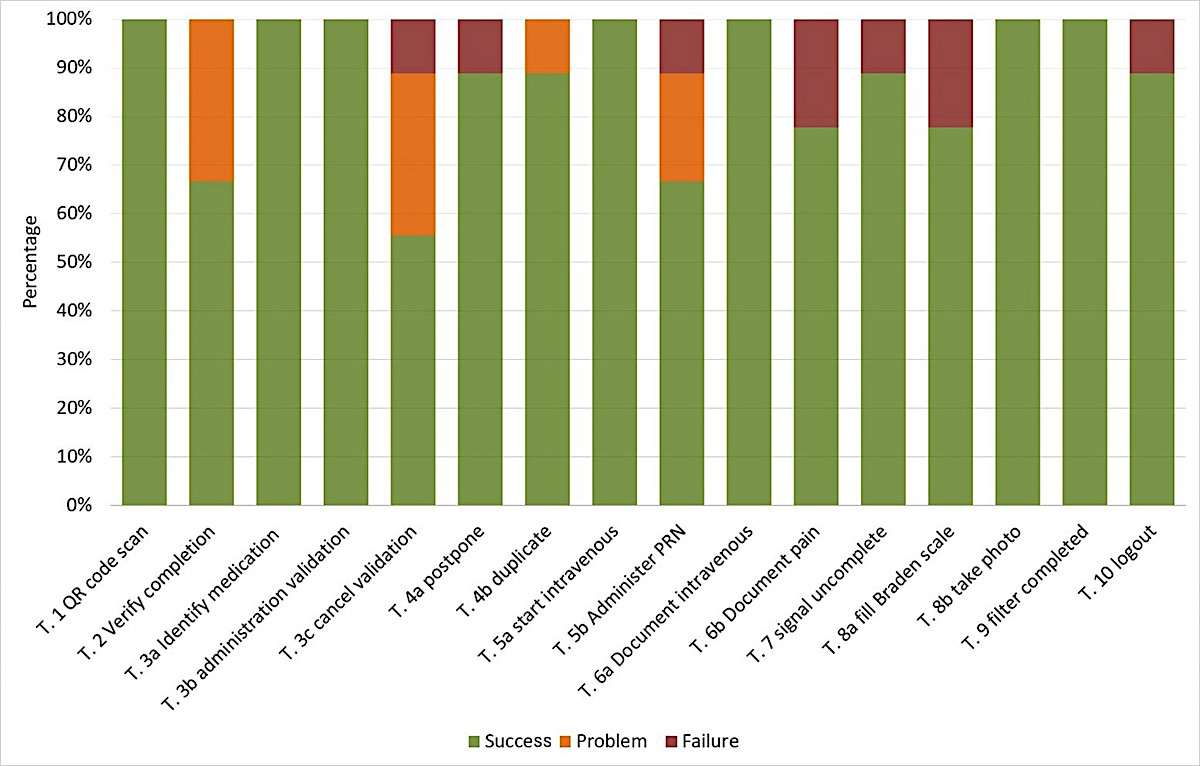
Success rates for task completion (n=9).

The test session for P1 did not record properly and was not included in the video analyses. Therefore, only the data of 9 participants could be analyzed for the goal-oriented tasks. For the analyses, the results of both scenarios were summarized. They are presented in the sequence of the surgical scenario. Figure 2 gives an overview of the success rates for all tasks of the evaluation.

### Task 1—Patient Identification

The first task, which was to select the patient by scanning the QR code, was completed successfully by all participants. Two nurses had problems while scanning the code due to the orientation of the camera during the scan. Since this is more a camera usability issue rather than an app issue, we did not code this as a usability issue. Another participant misunderstood the instructions. He initially attempted to select the patient through the manual selection (unit—room—patient) and not with the QR code. After the test manager advised him to reread the task description, he scanned the code without problems. However, before scanning the QR code, he returned to the home screen of the app, even though that function was available on the screen he was on.

### Task 2—Interventions Completion Control

**Figure 3:**
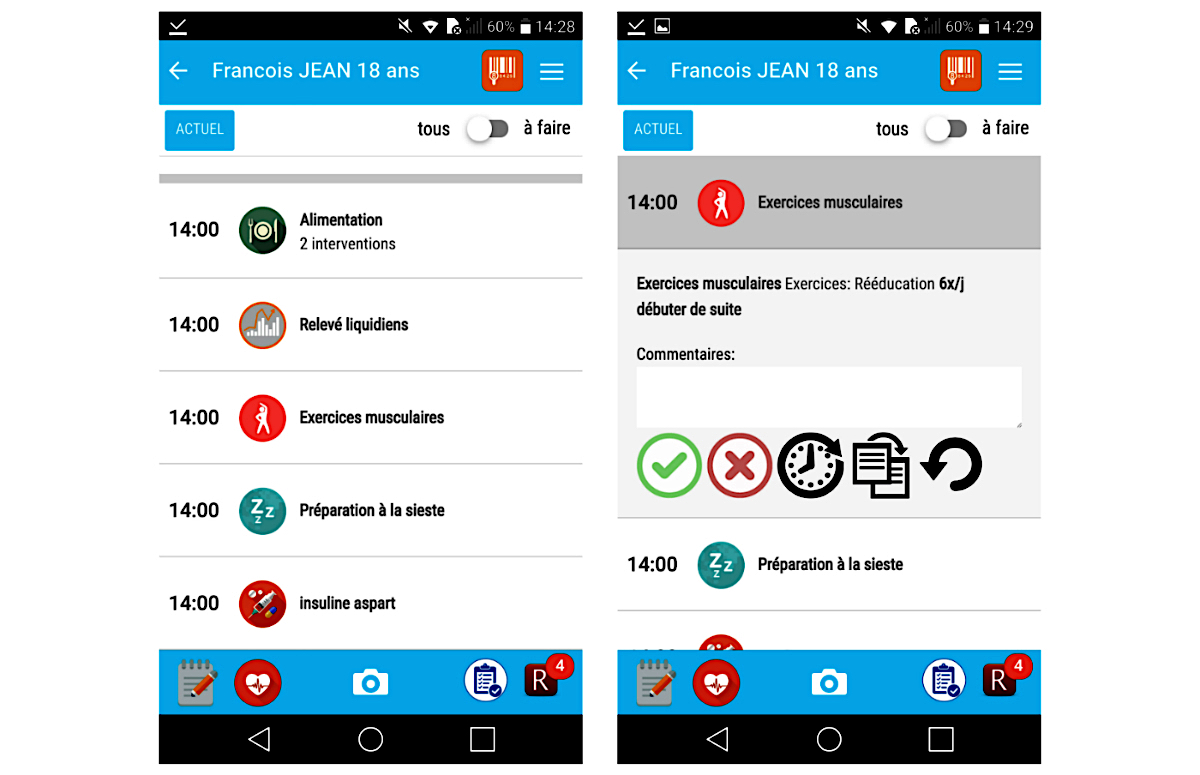
Problem related to use of back button. Whereas an opened intervention is closed by clicking on it again, many users used the back button and returned on the previous screen (patient selection screen).

The second task was to verify the correct completion of interventions performed during the previous shift. Although all participants managed this task, 3 participants had problems with the use of the “back button” (see Figure 3). Indeed, clicking an intervention opens an accordion that displays advanced interaction options. The participants attempted to close this accordion using the back button, which led them back to the previous screen. Participants then had to rescan the bracelet and therefore took longer to complete the task.

### Task 3—Medication Round

The third task consisted of three actions. First, the participants had to identify the interventions associated with the medication round (Task 3a). Then, they had to validate the administration of the drugs in the app (Task 3b). Finally, they had to cancel the validation of a meal intervention that had been previously validated by mistake (Task 3c).

**Figure 4:**
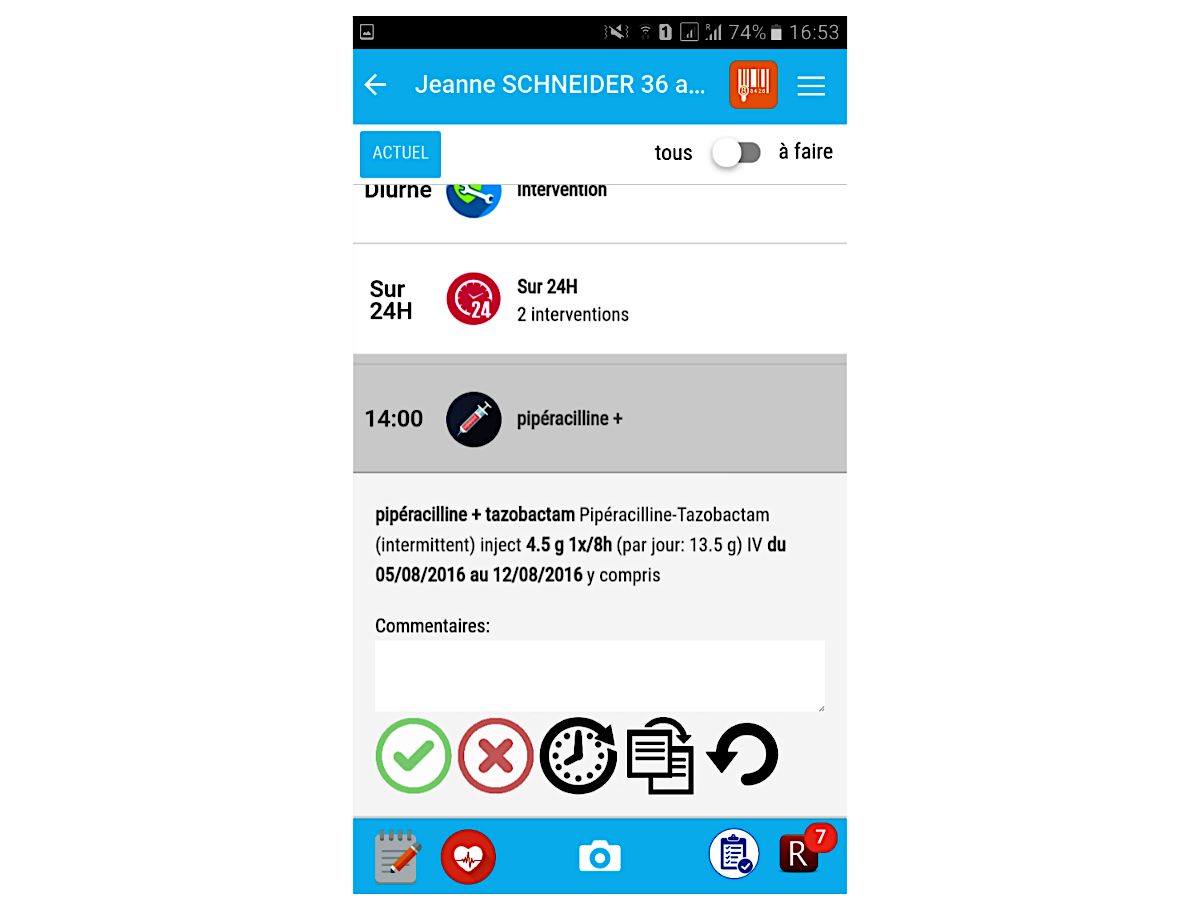
Problem with distinction of icons. The red “X” button is used to document an incomplete intervention and the “rounded arrow” button is used to undo the validation of a task.

The first subtask (Task 3a) was completed by all nurses without any problems. Two nurses chose a suboptimal approach for the second subtask (Task 3b): although the swiping validation is quicker, a task can also be validated via an icon that appears when clicking on the intervention. The two oldest participants, who also have the least experience with smartphones and mobile apps did not recognize this possibility, yet had no trouble validating via the icon. Three other participants had difficulties with the third subtask (Task 3c). They mixed up the icons for validating an uncompleted task and for undoing the validation of an intervention (see Figure 4). Initially, they clicked on the red icon with the cross, but this validates that the intervention was not completed rather than undoing the validation. One nurse recognized this mistake herself. The other 2 participants only corrected this error after the test manager asked them whether they were sure about their correct completion of this task. One participant was not able to solve the last subtask at all. First, he unintentionally pressed the home button of the smartphone and closed the app. After opening it again, he could not find an option to remove the validation of the intervention.

**Table 1:** Demographics of the study participants (n=10).

Characteristics		n (%)
**Age**		
	21-30 years	3 (30)
	31-40 years	5 (50)
	41-50 years	2 (20)
**Gender**		
	Female	6 (60)
	Male	4 (40)
**Professional experience**		
	< than 1 year	0
	1-5 years	4 (40)
	>than 5 years	6 (60)
**Experience with the CIS^a^**		
	< than 1 year	
	1-2 years	3 (30)
	>than 2 years	7 (70)
**Type of personal smartphone**		
	iOS	5 (50)
	Android	4 (50)
	Nokia	1 (10)
**Frequency of mobile apps use**		
	Often (daily)	8 (80)
	Regularly (several times per week)	
	Sometimes (1 to several times per month)	1 (10)
	Rarely (1 to several times per year)	
	Never	1 (10)

^a^CIS: clinical information system.

### Task 4—Postponing and Duplication

All nurses except one managed to postpone (Task 4a) and to duplicate (Task 4b) an intervention requested in Task 4. Instead of postponing the intervention, the nurse made a copy of the intervention. Another user navigated out of the patient chart after clicking on the wrong button (see descriptions of results for Task 1). But after reopening the patient data, he performed all subtasks without problems.

### Task 5—PRN Medication

**Figure 5:**
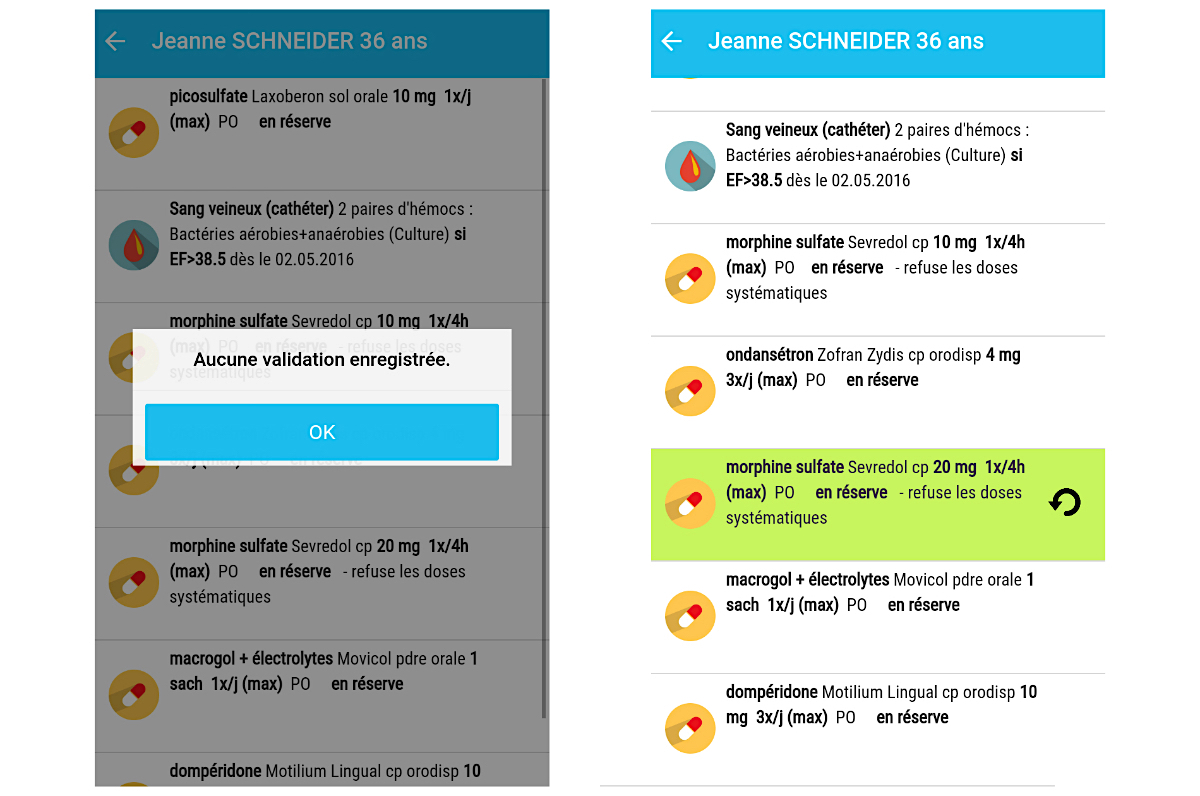
Problem with administration/validation of drugs from reserve.

To complete Task 5, nurses had to begin an intravenous medication for the patient and document in the app (Task 5a). Furthermore, as described in the scenario, the patient complained of pain during the administration. The nurse was then supposed to look up which PRN painkiller the patient had before administering a dose of that drug and validating this action (Task 5b). Three participants had problems with the completion of this task. Contrary to the interventions that had to be validated in Task 3, the administration of a PRN medication can only be validated via the swipe gesture (clicking on an entry opens the history of administration; see Figure 5). One nurse was not able to validate the drug administration at all. The other 2 participants needed more time to find this solution. However, the participants facing difficulties with this task were the same as those who did not use the swiping validation for the completion of Task 3.

### Task 6—Documentation of Pain Level

Task 6 was related to the previous one. After documenting the PRN painkiller (Task 6a), the participant was asked to document the pain level in the app (Task 6b). Two participants were not able to complete the second subtask. This action is accessible via an icon (“clinical scale”) in the footer bar of the app. However, those nurses were not able to link this icon with the associated functionality (see Figure 4, second icon from right).

### Task 7—Documentation of Uncompleted Intervention

Task 7 was performed successfully by all nurses except for one. He did not understand that intervention could be flagged as incomplete. Instead, he validated the intervention as done and entered a free-text comment indicating that intervention was incomplete because the patient refused to eat.

### Task 8—Braden Scale and Photo

For Task 8, participants were asked to take a photo of a red lesion on the patient’s elbow (Task 8a) and to fill out a Braden scale (Task 8b) to report the risk of pressure ulcers. This scale was accessible through the same icon used for the documentation of the pain level for Task 6. All participants were able to take the photo without difficulties. However, the 2 users who did not manage to document the pain level did not succeed to document the Braden scale either.

**Table 2:** Task completion by participants (S=Success, P=Problem, F=Failure).

Tasks	P2	P3	P4	P5	P6	P7	P8	P9	P10	%S	Nb P	Nb F
1. Patient Identification	S	S	S	S	S	S	S	S	S	100	0	0
2. Reading interventions	P	S	P	S	P	S	S	S	S	67	3	0
3A. List drug interventions	S	S	S	S	S	S	S	S	S	100	0	0
3B. Validate drug interventions	S	S	S	S	S	S	S	S	S	100	0	0
3C. Cancel intervention	S	S	S	P	P	P	F	S	S	56	3	1
4A. Postponing	S	S	S	S	S	S	F	S	S	89	0	1
4B. duplication	S	S	P	S	S	S	S	S	S	89	0	0
5A. Starting infusion intervention	S	S	S	S	S	S	S	S	S	100	0	0
5B. Reserve administration	P	S	S	F	S	S	F	S	S	67	2	1
6A. Ending infusion intervention	S	S	S	S	S	S	S	S	S	100	0	0
6B. Documentation of pain level	S	F	S	S	S	S	F	S	S	78	0	2
7. Signal incomplete	S	S	S	S	S	S	F	S	S	89	0	1
8A. Fill Braden scale	S	F	S	S	S	S	F	S	S	78	0	2
8B. Take a photo	S	S	S	S	S	S	S	S	S	100	0	0
9. Filtering interventions	S	S	S	S	S	S	S	S	S	100	0	0
10. Log out	S	S	S	S	S	S	F	S	S	89	0	1
Task success rate (%S)	88	88	88	88	88	94	56	100	100			
Total problem (Nb P)	2	0	2	1	2	2	0	0	0			
Total failure (Nb F)	0	2	0	1	0	0	7	0	0			
IOS (I), Android (A), Nokia (N)	I	I	A	I	A	I	A	A	N			

### Task 9—Filtering Interventions

Task 9 was to filter the completed interventions, keeping only the pending tasks visible during their work shift. This task was completed successfully by all participants.

### Task 10—Validation of Open Interventions and Logout

The last task of the test was to perform a logout. All except one participant managed through this task. He unintentionally clicked on the “back” icon and was dropped out of the patient screen. He did not try to disconnect after that. Instead, he gave feedback about the design of several icons, which were not clear for him.

Table 2 provides an overview of the success rates of the individual participants for the different tasks. “S” (success) means that the user solved the task easily. “P” (problem) indicates that the user had a problem but finally managed to complete the task. “F” (failure) indicates uncompleted tasks.

### Time on Task

The 2 evaluators differed on their measurement of duration for more than 10% on 25.7% (37/144) of the observations. The inter-rater agreement was high with a score of 0.976 obtained using the Krippendorff alpha test.

**Figure 6:**
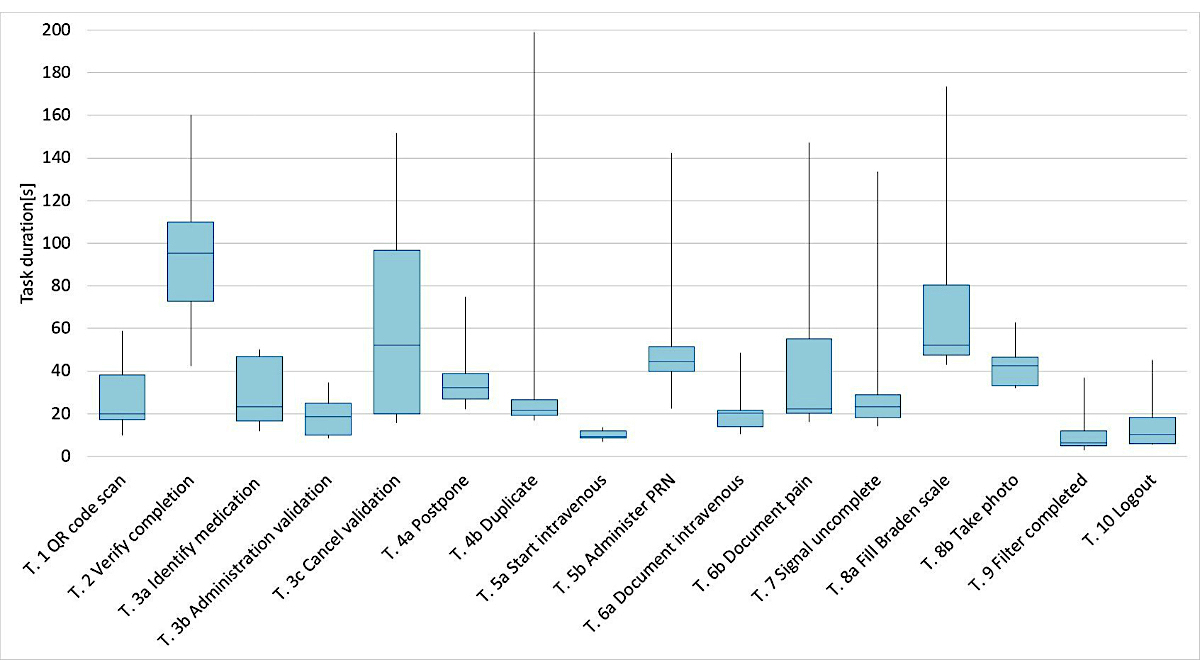
Boxplot of time spent on each tasks.

Regarding the time spent on the different task, we observed (Figure 6) that Task 2, which was requesting to review the completion of previous interventions, took the longest time with a mean of 94.4 s. Task 8a, which consisted of completing the Braden scale, also took a long time, with an average of 72.1s. Finally, Task 4b regarding duplication took a very long time for participant 4 because he first postponed the task instead of duplicating it.

### Perceived Usability and Satisfaction

The results of the SUS score are displayed in Table 3.

**Figure 7:**
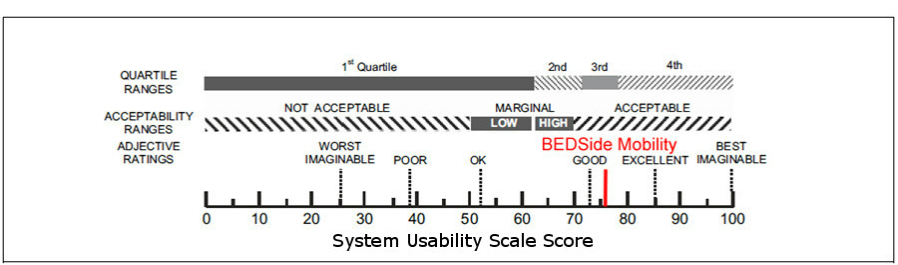
Adjective rating of BEDSide Mobility app.

As visible in Table 3, the app was rated with a mean average score of 76.3 (SD 16.75). According to Bangor et al (2009), a mean SUS score of 71.4 or higher can be interpreted as good [32]. Sixty percent (6/10) of our participants rated the BEDSide Mobility app as good, 20% (2/10) assessed it as excellent, and only 20% (2/10) rated it as okay (10%, 1/10) or poor (10%, 1/10). The adjective rating of the app is shown in Figure 7.

Forty percent of the participants completed all tasks without any problem. Thirty percent of participants had difficulties with one subtask but were finally able to accomplish all tasks. Only 2 users (P3 and P8) had problems with more than one task. In the SUS score, participants rated the app as good usability overall. It is not surprising that the participants who gave the lowest SUS ratings (P8 and P10) also had the most difficulties during the goal-oriented tasks.

Tasks 6b and 8a (Braden scale and pain level scale) both required accessing the clinical scale screen via an icon on the foot menu of the app and was not completed by P3 and P8. Either the icon used to represent this function was not comprehensible for some participants, such as P3, or the participants’ mental models for documentation did not correlate well with the design of the app.

The most difficult tasks for the participants were Tasks 2 (review interventions), 3c (cancel intervention) and 5b (validate PRN medications). The issue with Task 2 was related to the navigation in the app—clicking on the intervention opens an accordion to see advanced functions and details. Users tended to click the back button to close the accordion, but it actually led them back to the previous page.

**Table 3:** Results of System Usability Scale (SUS).

Questions	P1	P2	P3	P4	P5	P6	P7	P8	P9	P10	Average	Mean
1. I think that I would like to use this system frequently	3	4	3	3	4	1	2	2	4	2	2.8	3.0
2. I found the system unnecessarily complex	4	4	4	2	4	4	2	2	4	3	3.3	4.0
3. I thought the system was easy to use	4	4	3	3	4	3	4	2	4	2	3.3	3.5
4. I think that I would need the support of a technical person to be able to use this system	4	1	3	3	4	4	4	1	4	3	3.1	3.5
5. I found the various functions in this system were well integrated	3	2	3	3	3	4	1	1	4	2	2.6	3.0
6. I thought there was too much inconsistency in this system	3	4	4	3	4	4	2	1	4	4	3.3	4.0
7. I would imagine that most nurses would learn to use this system very quickly	2	3	2	4	2	0	4	1	3	3	2.4	2.5
8. I found the system very cumbersome to use	3	4	4	3	4	4	4	0	4	3	3.3	4.0
9. I felt very confident using the system	4	3	2	3	4	2	2	2	3	2	2.7	2.5
10. I needed to learn a lot of things before I could get going with this system	4	4	3	3	4	4	4	2	4	3	3.5	4.0
SUS-score (sum × 2.5) [maximum 100]	85.0	82.5	77.5	75.0	92.5	75.0	72.5	35.0	95.0	67.5	75.8	76.3

**Table 4:** Identified shortcomings and correction measures.

Identified shortcomings	Correction measures
Miscomprehension of the clinical scale icon	Identification with the users of a more appropriate icon to represent clinical scale
Unexpected navigation of the back button when an intervention is open	Modification of the navigation mechanism by closing the intervention when opened rather than returning to the previous page
Canceling the validation of an intervention	Improved explanations before app use can help avoid this confusion
Inconsistent implementation of the functional design validating the administration of a PRN^a^ drug	Integration of similar validation mechanism to administer PRN drug using consistent icons

^a^PRN: pro re nata.

One participant remarked:

In fact, the mistake is that I clicked there to go back [the user points to the arrow icon (back button)]. You just have to click above.

P4

Overall, the evaluation results and SUS scores show that the tested prototype of the app already has a good usability. Correction measures were identified to address the shortcomings (Table 4). These correction measures did not only involve modifying the interface but also have implications for the deployment and user training at that time.

## Discussion

### Principal Findings

The purpose of this study was to assess the usability of the BEDSide Mobility app, which can inform the development of similar tools in other settings. A previous study utilized a heuristic review to identify usability issues before this user study, which were then subsequently fixed. This study is aimed at identifying further issues, as users may have unexpected issues or problems that were not anticipated by the designers of the app.

While participants rated the app as having good usability overall, there are some issues that can be fixed to improve the experience for users and hopefully help a greater number of users complete their specified tasks smoothly. The participant who gave the lowest SUS score had the most difficulty completing the tasks and also happened to be the oldest participant in the study (P8). This participant also reported a low use of mobile apps outside the study, suggesting that it was probable that the failure of task completion was partly due to a lack of familiarity with mobile apps, complaining that:

It's the app that is not logical. It is not logical.

This participant also gave a much lower rating of the app with SUS than all others (see Table 3).

Such reactions suggest that designers should take into consideration the wide variance of people who could be using the app, from people who have a high familiarity with mobile app conventions and use to those who have very little familiarity with technology and mobile app conventions. These two populations are often correlated, with older adults showing lower mobile adoption than younger adults. This observed resistance is also in line with previous findings in the literature, which show that low familiarity with computer and older age are barriers to the adoption of new technologies [33]. As such, if the said population constitutes a sizable amount of the users that may use any future apps, extra care should go into education and easy learnability of the app by both tutorials and best practices, depending on the makeup of the potential users.

Some participants had issues linking functionality to certain iconography within the app. For example, one-third of the (n=3) participants had problems with distinguishing the icons related to tagging an intervention as incomplete versus undoing a validation of an intervention. Also, a number of associated participants had trouble identifying the icon that would allow data collection via a clinical scale. If designers wish to maximize the number of users who can easily pick up the app and use, iconography should be tested with the target population. However, some more complex workflows may not be able to work only with icons, and text labels could greatly improve the ease of use of the app. Alternative methods could include education or a quick tutorial to see whether the icon makes sense after pointing out the functionality behind it.

Having an easy undo to actions could also encourage users to explore the interface more since they know there would be a quick way to undo any action if they take the wrong action. We recommend implementing such functionality so that users are encouraged to use the app with minimal chance for permanent errors.

The consistency of actions design is also an important way to keep users happy with the workflow. For example, issues with Task 5b were caused by an inconsistent implementation of the functional design. In general, all interventions in the app can either be validated by using a specific icon or by a swiping gesture. However, validating the administration of a PRN drug is only possible by swiping since there is no icon in that dialog. By changing the actions available, participants had issues completing the task. Future designers should clearly identify what functionality and interface actions they wish to support and keep it consistent throughout the whole app to facilitate ease of task completion.

Our findings are consistent with existing guidelines, previous studies, and recommendations. For example, previous guidelines recommend allowing user control and freedom with easy undo, to have consistency and standards, and have users use recognition rather than recall in the interface to minimize memory load. While these guidelines are a great place to start, for maximum use and usability, extensive user testing should take place throughout the design process to make sure that the app will match user’s mental model and to have them use an easy, intuitive app that meets their needs.

### Limitations

With regard to the results of the study, two main limitations have to be noted. On the one hand, a sample of 10 nurses may be insufficient to reveal all usability issues. However, previous works with 9 to 10 participants have shown good cost efficiency and should allow most of the usability problems to be identified [7,8,34]. On the other hand, we used an artificial lab environment, which has a low degree of fidelity. We simulated the patient with a static mannequin, and the environmental influences such as noise, interruptions from patients or other colleagues, etc, have been eliminated. Therefore, the generalizability and transferability of the results may be limited in a real setting.

### Conclusions

This study aimed to assess the usability and the suitability of the BEDSide Mobility app to facilitate the caregivers’ workflow at the patient’s bedside. Our study identified several usability flaws. Among them, the navigation incoherence, in particular, was cumbersome during use and should be corrected in priority. Some inconsistencies in the design were barriers to the successful completion of some tasks. Other problems were linked to the lack of clarity of some icons and their associated functionalities. This can be improved by choosing better signalization. If the interface can be improved to mitigate some issues, appropriate training, and deployment measures should also be implemented to avoid misuse of the app. It was reassuring that no data entry problems occurred during our study, as this can be an important source of errors.

Besides the problems identified, the results indicate a good usability, a satisfactory acceptance, and satisfaction of the participants with the developed app. This tends to demonstrate the relevance of our end user-centered approach in the development of tools dedicated for care providers. Indeed, end users were involved in formative evaluation rounds throughout the specification and development phase to minimize the gap between their requirements and the actual realization.

Finally, it is important to recognize that the ecological validity of the experimental setting was quite low. Therefore, additional usability flaws may occur when the tool is used in the real setting. This study is a part of a more global assessment of the efficiency of the app that will be tested in a real setting.

## Abbreviations

CIS: clinical information system
COWs: computers on wheels
EHR: electronic health record
F: failure
P: problem
PRN: pro re nata
QR: quick response
S: success
SUS: System Usability Scale
